# A hybrid method combining rule-based filter and machine learning to detect porpoise and vessel sounds from a pulse event recorder

**DOI:** 10.1038/s41598-025-16370-1

**Published:** 2025-08-25

**Authors:** Mayu I. Ogawa, Satoko S. Kimura, Nozomu Ishiai, Tomonari Akamatsu

**Affiliations:** 1 Underwater Biological Sound Analysis Group, Smart Sensing Technology Development Center, Research Institute for Marine Technology and Engineering, Japan Agency for Marine-Earth Science and Technology (JAMSTEC), Yokosuka, Kanagawa Japan; 2 Graduate School of Agriculture, Kyoto University, Kyoto, Japan; 3 Center for Southeast Asian Studies, Kyoto University, Kyoto, Japan; 4 Research Organization for Nano & Life Innovation, Waseda University, Shinjuku, Tokyo, Japan

**Keywords:** Passive acoustic monitoring, Odontocetes, Finless porpoise, Ship noise, Random forest, Computational biology and bioinformatics, Ecology

## Abstract

Passive acoustic monitoring is essential for assessing the impact of anthropogenic noise on marine ecosystems and detecting vocalizing marine life. While acoustic event recorders are widely used to record odontocete echolocation due to their low power and memory demands, conventional detection algorithms are often unsuitable for analyzing datasets composed of complex pulse events. Here, we developed a hybrid analytical framework combining a rule-based filter with a random forest model to efficiently detect narrow-ridged finless porpoise (*Neophocaena asiaeorientalis*) click trains and vessel noise events using data from the pulse event recorder. The rule-based filter effectively reduced noise from raw data, achieving detection accuracy of almost 100% for click trains and 94% for vessel noise. However, among the events detected by this filter, 45% and 81% were actually false positives. The machine learning model improved classification accuracy to 97% and 99%, respectively. This model reduced the high false positive rates to 2.8% and 0.1%. This combined method offers a robust and efficient approach to processing pulse event recorder data, specifically for A-tag. It reduces manual workload, improves detection accuracy, and facilitates rapid assessment of vessel noise impacts, thereby supporting long-term ecological monitoring of small cetacean populations in diverse and noisy marine environments.

**Supplementary Information:**

The online version contains supplementary material available at 10.1038/s41598-025-16370-1.

## Introduction

The underwater environment contains various acoustic sources, commonly categorized as geophony (e.g., earthquakes and rainfall), biophony (e.g., vocalization of whales and fish), and anthrophony (e.g., vessel noise and pile driving sound)^1,2^. Analysis of underwater soundscape data enables biodiversity assessment and enhances understanding of species occurrence patterns through the detection of acoustic signals from cetaceans and soniferous fish^3–7^. Acoustic monitoring and analytical techniques are also increasingly used to evaluate the impact of anthropogenic sounds, such as vessel noise, on marine life^8–13^. Therefore, efficient and accurate methods for detecting these acoustic sources and assessing their ecological impacts are needed.

Passive acoustic monitoring is a powerful tool for detecting and measuring underwater acoustic signals^5,14^. Autonomous acoustic recorders enable continuous monitoring until battery or memory capacity is exhausted. Echolocation click trains produced by small odontocetes contain ultrasonic components up to 150 kHz^12^, necessitating high sampling frequencies, which pose challenges for long-term monitoring^15^. To address this issue, ultrasonic pulse event recorders such as F-POD^16–18^ and A-tag^15^ have been developed. The A-tag, specifically designed for long-term recording of odontocete click trains, has been widely adopted in odontocete monitoring^19,20^. Pulse event recorders require less memory capacity since they store the intensity, event time, and associated parameters such as band intensities without recording the entire waveform. Power spectrum characteristics of click trains show only limited differences among species^12^. Off- or on-axis effects significantly change the received spectrum shape^21^. Therefore, recording the full waveform is not essential for detecting small odontocetes. By omitting waveform recording, the A-tag achieves low power consumption and efficient memory storage.

Besides these benefits, a major challenge in analyzing pulse event recorder data is that conventional detection and identification algorithms are not applicable. To address this, Kimura et al.^20^ developed a rule-based filter to extract click trains from the A-tag data for Yangtze finless porpoise (*Neophocaena asiaeorientalis asiaeorientalis*) under relatively silent river conditions. The rule-based filter refers to set of analysis codes that apply multiple criteria to time-series pulse event data to detect clusters of pulse corresponding to target events. However, many dolphin and porpoise species inhabit noisy marine environments due to biological sources, such as snapping shrimp.

To overcome these challenges associated with noisy marine environments, we developed a hybrid approach combining a rule-based filter and a machine learning model to extract target acoustic events and minimize false alarms in the pulse event recording data. We applied this method to identify click trains of narrow-ridged finless porpoise (*Neophocaena asiaeorientalis*) and vessel noise events in Japanese coastal waters, where intense biological and anthropogenic noise often masks target signals. Manual detection in such environments is often time-consuming. However, our approach enables efficient and automated detection, offering a practical solution for long-term monitoring in acoustically complex environments.

## Materials and methods

### Monitoring sites and periods

**Fig. 1:**
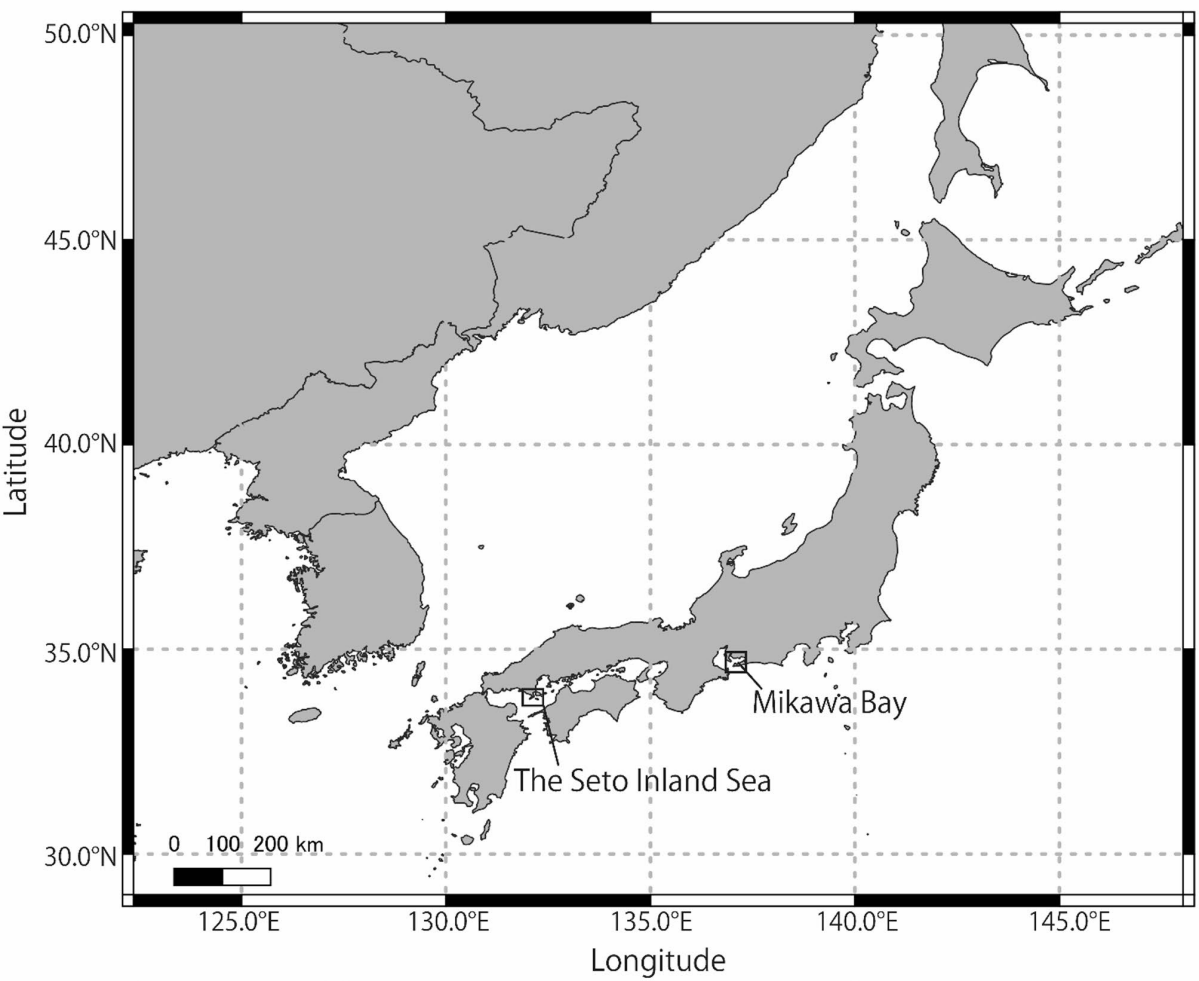
Locations of two data collection areas. The data for training and validation of machine learning model were recorded in Mikawa Bay. The data for testing effectiveness of the developed model were recorded in the Seto Inland Sea. The area enclosed by the rectangle indicates the recorded area. Maps were created in QGIS 3.40.6^26^ with administrative boundaries from GADM^27^ and coastline data from Natural Earth Data (https://www.naturalearthdata.com/).

Data for developing the rule-based filter and machine learning model were collected in Mikawa Bay, Japan, while additional data were obtained from the Seto Inland Sea, Japan, to test the effectiveness of the developed algorithm (Fig. 1). The data were intermittently collected from October 2013 to December 2023 in Mikawa Bay (Table S1). Test data were collected from July 2021 to December 2023 in the Seto Inland Sea, a geographically separated region from Mikawa Bay (Fig. 1, Table S1). This area hosts a distinct finless porpoise population compared to that in Mikawa Bay^22–24^ and exhibits different background noise levels^25^. Incorporating such variation into the test data enables evaluation of the model’s robustness to minor differences in click train characteristics across sites, populations, and acoustic backgrounds^25^.

All methods were non-invasive and carried out in accordance with relevant guidelines and regulations. The Kyoto University Animal Experiments Committee approved experiments (Inf-K15003, Inf-K16002, Inf-K17004, Inf-K18004, Inf-K19004, Inf-K20010, Inf-K21008, 1–202202, 1–202302). This study is reported in accordance with the ARRIVE guidelines.

### Data acquisition instruments

We used A-tags^15^ (MMT, Saitama, Japan) as pulse event recorders designed to monitor high-frequency underwater acoustic signals. Each A-tag comprises a stereo hydrophone system (hereafter, hydrophones A and B), preamplifier with bandpass filter, CPU, flash memory, and two alkaline batteries. Hydrophone A and B have peak sensitivities at 70 and 130 kHz, respectively^28,29^ (Figure S1). Two configurations were used: T-type with horizontally arranged hydrophones spaced 590 mm apart, and an I-type with vertically aligned hydrophones spaced 190 mm apart (Figure S1). Since no substantial difference was found in detecting target pulse events between these configurations, the same analysis procedures were applied to all data. The A-tag detects ultrasonic pulse events within the 55–235 kHz band, and records the time of detection of each pulse, the received sound pressure levels (SPL) at the two hydrophones, and the time of arrival difference between the two hydrophones only when the received pressure exceeded a predefined amplitude threshold. Unlike other event recorders, which classify detected signals onboard and store only the processed results^16–18^, the A-tag preserves raw pulse event parameters without onboard classification. Pulse event was stored with a minimum time resolution of 0.5 ms, with the detection threshold set at 139 dB re 1 µPa. The time difference of arrival between the two hydrophones was measured at a resolution of 0.25 µs and stored in association with the time of detection (0.5 ms resolution) and received SPL of both hydrophones. The SPL ratio (SPLR) between hydrophones A and B was used to infer the spectral characteristics of incoming pulse events, such as the relative proportions of high- and low-frequency components, which are useful for distinguishing between Phocoenidae and Delphinidae families^28,29^. The A-tag was deployed by suspending it from a buoy tethered by a rope, maintaining hydrophone A at a depth of 3 m.

### Target pulse events for detection

**Fig. 2:**
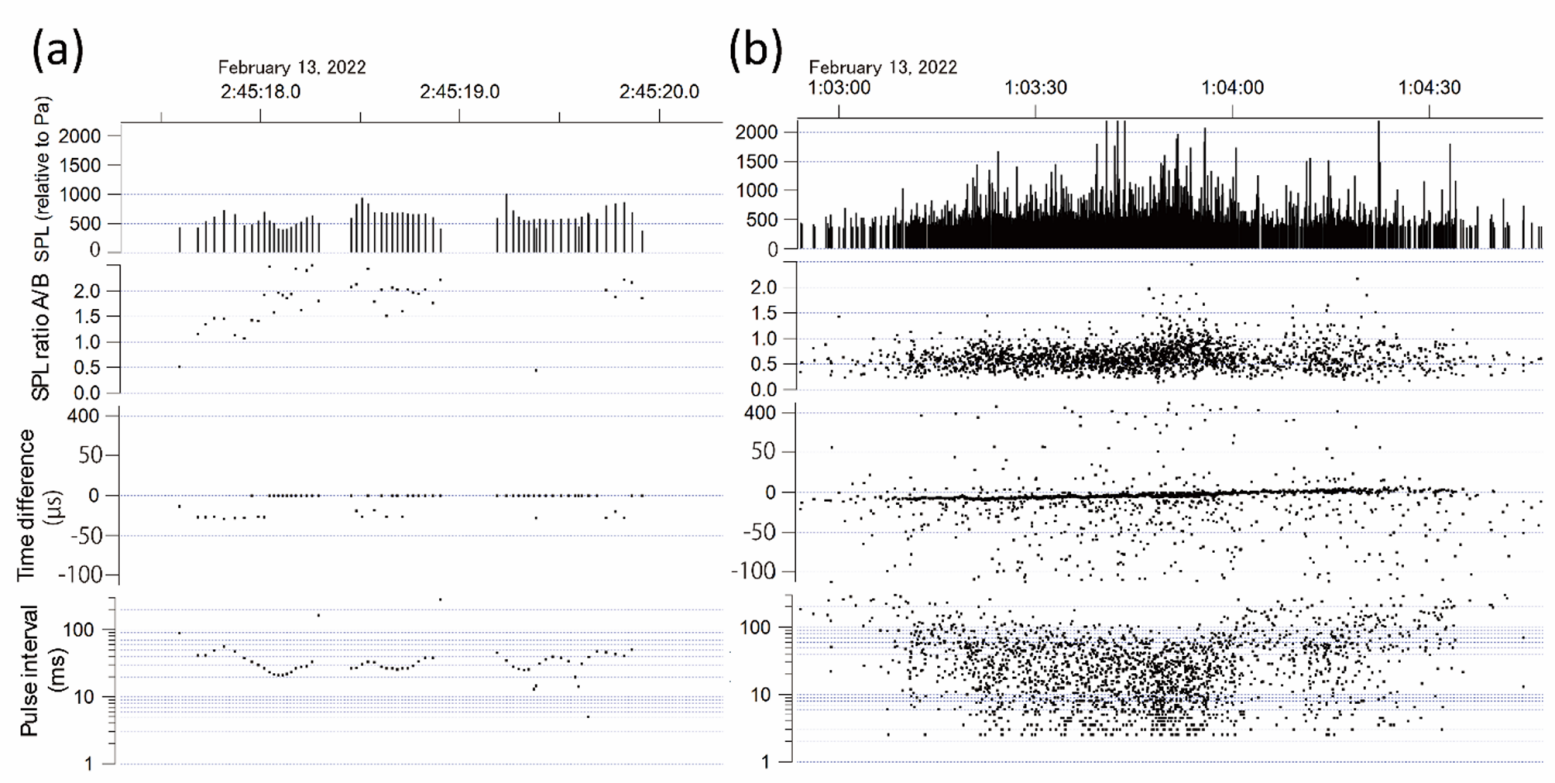
Examples of (**a**) finless porpoise click trains and (**b**) vessel noise recorded by the A-tag. The x-axis shows the recording time. The y-axes show the received sound pressure level (relative to Pa) at hydrophone A (SPL), the sound pressure level ratio between hydrophones A and B (SPL ratio A/B), the time difference (µs) between hydrophones A and B that can be converted to relative azimuth, and the pulse interval (ms). A positive value for the time difference indicates that the signal arrived at hydrophone A earlier than at hydrophone B.

This study aimed to detect acoustic events of click trains produced by the narrow-ridged finless porpoise. Click trains recorded by the A-tag exhibit smooth changes in SPL and pulse interval ^30,31^ (Fig. 2a). Unlike many delphinid species, finless porpoise produces only click trains with high frequencies ranging from 100 to 150 kHz. Their click trains were classified into two types: regular clicks, which are used for echolocation, and buzzes. Buzzes, characterized by pulse intervals of ≤ 10 ms, are typically produced during close-range prey approaches^32^.

This study also targeted high-frequency vessel noise that falls within the auditory sensitivity range of the finless porpoise^33,34^, as assessing noise impacts requires capturing sounds that porpoises can actually perceive. The A-tag detects pulse events only in the 55–235 kHz range, encompassing the auditory sensitivity peak (70-80 kHz) of finless porpoise^33,34^, and its therefore suitable for assessing high-frequency vessel noise within their ultrasonic acoustic environment. The detected vessel noise is characterized by irregular pulse intervals, SPLs, and time differences of arrival between the two hydrophones, and typically exhibits a prolonged duration^35^ (Fig. 2b). Such high-frequency noise, typically generated by small, high-speed vessels not equipped with AIS due to cavitation or other mechanisms associated with propellers and engines^36,37^, is presumed to originate from vessels passing in close proximity to the A-tag, as high-frequency sound attenuates rapidly and does not propagate over long distance.

### Overview of the development of a rule-based filter and machine learning model

**Fig. 3:**
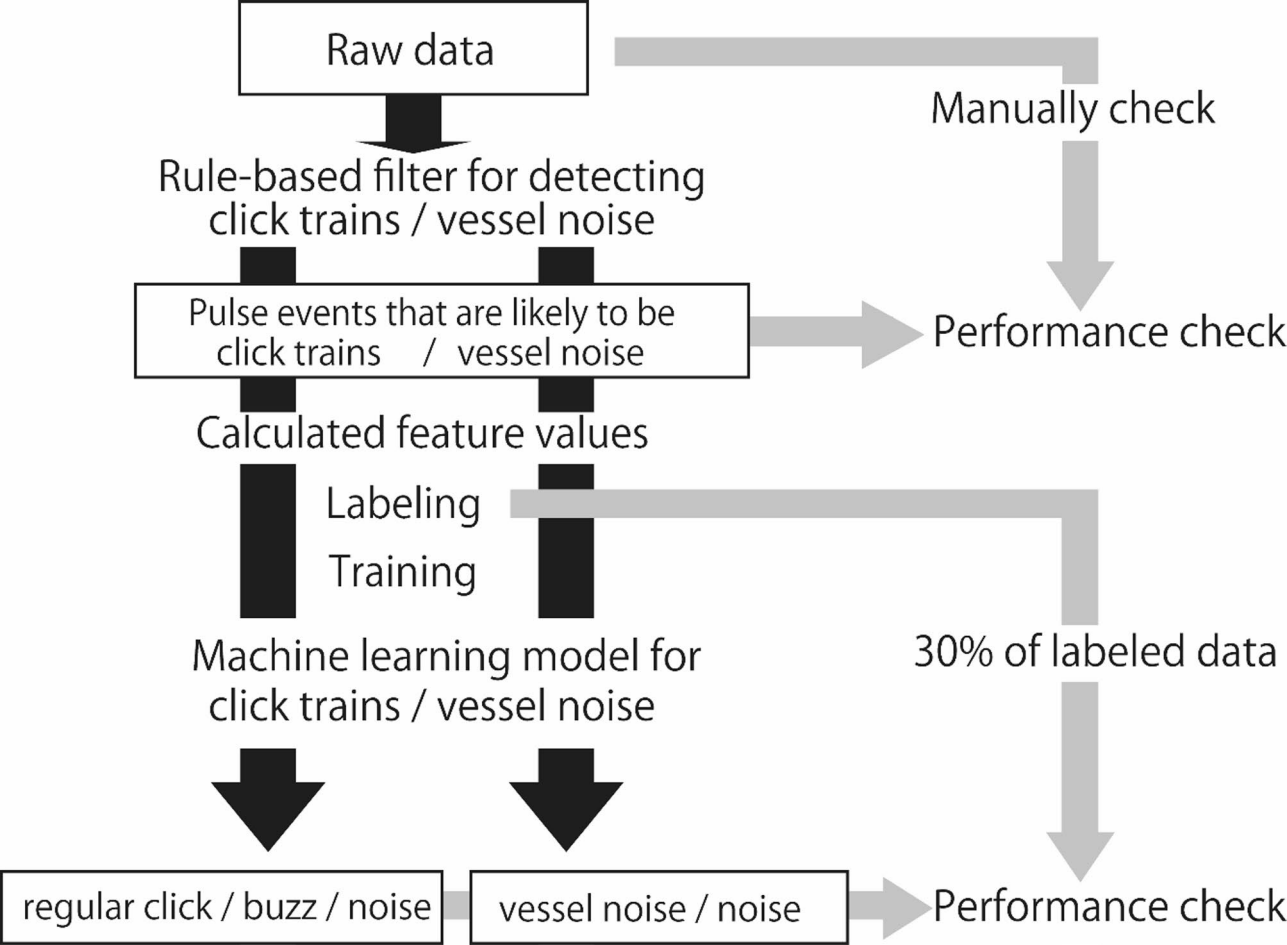
Flowchart of the development and validation processes of the rule-based filter and machine learning model used to classify finless porpoise click trains and vessel noise, based on training dataset.

This section outlines the development of the rule-based filter and machine learning model for detecting and classifying click trains and vessel noise. These two signal types differ markedly in their acoustic characteristics (Fig. 2). Due to these differences, each signal type was processed using a separate rule-based filter and a dedicated machine learning model (Fig. 3).

As a preprocessing step, separate rule-based filter was applied to raw data to eliminate irrelevant noise and extract pulse events likely to be either click trains or vessel noise (Fig. 3). The extracted pulse events were manually reviewed to evaluate detection performance.

For each detected pulse events, feature values were computed for training the corresponding machine learning model. Click-train-like events were labeled as regular clicks, buzzes, or noise, while vessel-noise-like events were labeled as vessel noise or non-vessel noise. Using these labeled events and their feature values, two machine learning models were trained: one to classify click train-related signals (regular clicks, buzzes, and noise) and the other to classify vessel noise events (vessel noise and non-vessel noise). The classification performance of each machine learning model was then evaluated using 30% of the manually labeled dataset, which was held out as validation data.

### Development of a rule-based filter

The rule-based filter for detecting click trains and vessel noise was developed using Igor Pro 64 8.04 (WaveMetrics, Portland, OR, USA). This filter was based on the detection criteria described by Kimura et al.^20^, originally developed to extract Yangtze finless porpoise from stationary A-tag recording. In that study^20^, the criteria included the following: a passive SPL threshold (≥ 140.4 dB re 1 μPa), a minimum pulse interval (≥ 2.0 ms), a maximum pulse interval (≤ 100 ms), at least six pulses per click train, and coefficient of variation of pulse interval ≤ 0.4. Of these criteria, this study adopted the following criteria for the rule-based filter, excluding the detection threshold, which was predefined by the A-tag’s recording setting: minimum pulse interval (≥ 2.0 ms), a maximum pulse interval (≤ 100 ms), a minimum of six pulses per click train, and coefficient of variation of pulse interval ≤ 0.4 (Table 1). Because click trains produced by Phocoenidae, including finless porpoises, typically exhibit an SPLR greated than 0.6^28,29^, a threshold of 0.6 was adopted for the rule-based filter (Table 1). Additional criteria were initially defined based on empirical knowledge. These criteria were subsequently refined by comparing filter outputs with manual annotations of the raw data, to improve click train detection. These criteria (Table 1) included the following: a minimum duration of click train (≥ 12 ms), a maximum standard deviation of arrival-time differences between hydrophones A and B (< 25 µs), a maximum coefficient of variation (standard deviation/mean) of received SPL at hydrophone A (≤ 100), and maximum median pulse interval within a click train (< 100 ms). Pulse events satisfying all nine criteria were selected as candidate finless porpoise click trains.

**Table 1:** Criteria used in the rule-based filter for detecting finless porpoise click trains.

No	Criteria for identifying finless porpoise click trains
1	Pulse intervals must be ≥ 2 ms to exclude reflections
2	Pulse intervals between clicks must be ≤ 100 ms
3	Click trains must contain at least six pulses
4	Coefficient of variation of pulse intervals (standard deviation/mean) of pulse intervals within a click train must be ≤ 0.4
5	SPLR must be ≥ 0.6
6	Click train duration must be ≥ 12 ms
7	Standard deviation of arrival-time differences between hydrophones A and B must be < 25 µs
8	Coefficient of variation (standard deviation/mean) of received SPL at hydrophone A within a click train must be ≤ 100
9	Median pulse interval within a click train must be < 100 ms

On the other hand, the rule-based filter for detecting vessel noise was developed using pulse intervals, number of consecutive pulses, and minimum continuous duration (Table 2), based on the typical acoustic characteristics of vessel noise. These criteria were optimized by comparing filter outputs with manual annotations of the raw data, resulting in final settings of pulse intervals shorter than 500 ms within pulse events, more than 80 consecutive pulses within a pulse event, and a minimum continuous duration of a pulse event of ≥ 10 s (Table 2).

**Table 2:** Criteria used in the rule-based filter for detecting vessel noise.

No	Criteria for identifying vessel noise events
1	Pulse intervals must be < 500 ms
2	Number of consecutive pulses must be > 80
3	Minimum continuous duration of a pulse event must be ≥ 10 s

The detection accuracy of the established rule-based filter was calculated using manually validated datasets. The raw time-series data recorded by A-tags were plotted using Igor Pro, manually annotated, and subsequently analyzed using the rule-based filter to evaluate the number of target pulse events (click trains or vessel noise) successfully detected within the validation dataset (Fig. 3). Detection rates were defined as the proportion of click trains or vessel noise events correctly identified by the rule-based filter relative to the total number manually confirmed in the raw data. Additionally, the total number of candidate events detected by the rule-based filter was also quantified. The accuracy of the rule-based filter for detecting click trains was evaluated based on a verified dataset totaling 36 h (Table S1). For vessel noise detections, a separate dataset was used due to the relatively low occurrence of such events, totaling 408 h (Table S1).

### Preparation of training and validation datasets for a machine learning model

Click trains and vessel noise events detected by the rule-based filter were characterized by 18 and 17 feature values, respectively. In addition to common acoustic parameters such as the number of pulses, duration, pulse intervals, and SPLs, temporal features represented by “Start” and “End” timestamps were also included (Table 3). These feature values were selected not only to capture seasonal and diel patterns, but also to incorporate empirical observations, such as that a pulse event is more likely to originate from finless porpoises if porpoise clicks have been detected immediately beforehand. Click trains were classified into regular clicks and buzzes following definitions established in previous studies, where a buzz was specifically defined as a sequence containing five or more consecutive pulses with intervals ≤ 10 ms^38,39^. To facilitate this distinction, a binary feature value called “BuzzCheck” was implemented to return 1 when the click train satisfied the definition of buzzes and 0 otherwise. The same set of feature values used for click trains was applied to vessel noise, excluding “BuzzCheck,” which was irrelevant for vessel noise.

**Table 3:** Feature values of pulse events as the training data.

Feature value name	Meaning
Np	Number of pulses in a pulse event
Duration	Duration of a pulse event
Start/End	Time (yyyy/mm/dd hh:mm) when the first and last pulse of a pulse event was recorded
MaxPi/MinPi/AvPi/SdPi	Maximum, minimum, average, and standard deviation of pulse interval within a pulse event
MaxSPLR/AvSPLR/SdSPLR	Maximum, average, and standard deviation of recorded sound pressure ratio (hydrophone A/B) within a pulse event
Maxtd/Avtd/Sdtd	Max, average, and standard deviation of arrival time difference between hydrophones A and B within a pulse event
MaxSPL A/AvSPL A/SdSPL A	Max, average, and standard deviation recorded sound pressure level of hydrophone A within a pulse event
BuzzCheck	Binary parameter indicating whether a pulse event from a finless porpoise contains five or more consecutive pulses with pulse intervals ≤ 10 ms; returns 1 if the definition of a buzz is met, and 0 otherwise

A subset of the training data was labeled for developing and evaluating machine learning models. After applying the rule-based filter, pulse events detected as click trains were manually classified and labeled into three groups: regular clicks, buzzes, and noise. In contrast, pulse events detected as vessel noise were manually classified and labeled into two groups: vessel noise and non-vessel noise. The labeling of click trains was based on a dataset totaling 72 hours, while labeling of vessel noise events was based on a dataset totaling 720 hours (Table S1).

### Development of a machine learning model

The machine learning model was developed using the random forest algorithm implemented in the scikit-learn toolbox in Python 3.9.7^40,41^. The random forest is a machine learning algorithm based on ensemble learning, in which multiple decision trees are combined to improve prediction accuracy^42^. Random forests are commonly used for classification tasks due to their robustness and high accuracy, particularly in classifying small cetacean vocalizations^43,44^. Training and validation were conducted using 70% and 30% of the labeled pulse event data, respectively (Fig. 3). Hyperparameters were optimized for each model, with a maximum tree depth of 30, a minimum sample split of 7, and a total of 100 estimators.

### Performance evaluation

The performance of the machine learning model was evaluated based on five metrics: accuracy (Eq. 1), precision (Eq. 2), recall (Eq. 3), F1-score (Eq. 4), and false positive rate (FPR) (Eq. 5). These metrics were calculated based on four values: true positive (TP), true negative (TN), false positive (FP), and false negative (FN). TP represents instances correctly predicted as positive, TN denotes instances correctly predicted as negative, FP refers to instances incorrectly predicted as positive, and FN refers to instances incorrectly predicted as negative. In this evaluation, regular clicks and buzzes were combined into a single category of click trains, based on definitions operationalized using the “BuzzCheck” feature. The binary feature enabled deterministic separation between regular clicks and buzzes, with no ambiguity or overlap. The performance of the machine learning model was evaluated separately for detecting click trains and vessel noise.

Accuracy indicates the overall ability of the model to correctly identify both target and non-target events. Accuracy was calculated using Eq. (1):

1 $$\begin{array}{*{20}c} {\frac{{{\text{TP}} + {\text{TN}}}}{{{\text{TP}} + {\text{TN}} + {\text{FP}} + {\text{FN}}}}} \\ \end{array}$$

Precision indicates the proportion of instances predicted as target events that were correctly classified. Precision was calculated using Eq. (2):

2 $$\begin{array}{c}\frac{\text{TP}}{\text{TP}+\text{FP}}\end{array}$$

Recall indicates the proportion of actual target events that were correctly identified by the model. Recall was calculated using Eq. (3):

3 $$\begin{array}{*{20}c} {\frac{{{\text{TP}}}}{{{\text{TP}} + {\text{FN}}}}} \\ \end{array}$$

F1-score indicates the harmonic mean of precision and recall and serves as a comprehensive metric for evaluating the balance between these two measures. F1-score was calculated using Eq. (4):

4 $$\begin{array}{c}\frac{2*\text{Recall}*\text{Precision}}{\text{Recall}+\text{Precision}}\end{array}$$

The FPR indicates the proportion of non-target events that were incorrectly classified as target events. The FPR was calculated using Eq. (5):

5 $$\begin{array}{c}\frac{\text{FP}}{\text{TN}+\text{FP}}\end{array}$$

Finally, to evaluate the time savings achieved by applying the developed rule-based filter and machine learning model, approximately 395 h of A-tag data were analyzed, and the total time required for manual detection was compared with that required when using the combined rule-based filter and machine learning approach.

### Validation of the developed algorithm on test data

To evaluate the generalizability of the rule-based filter and machine learning model developed using A-tag data recorded in Mikawa Bay, we evaluated them using a test dataset recorded in the Seto Inland Sea, separately assessing performance for click trains and vessel noise (Fig. 1). The performance of the rule-based filter was evaluated by comparing the number of click train and vessel noise events detected manually with those detected by the filter in the test datasets. Detection accuracy was calculated as the percentage of filter-based detections relative to manual detections. In addition, the total number of pulse events identified by the filter as candidate click trains or vessel noise was also counted. For the machine learning model, classification was performed on pulse events detected by the rule-based filter. The classification results were then compared with manual verification results to determine the number of TP, FP, TN, and FN instances. Based on these values, standard performance metrics—including accuracy, recall, precision, F1-score, and FPR—were calculated according to their respective equations. A total of 16 h of data were used for click train analysis, and 456 h for vessel noise analysis.

## Results

### Performance of the rule-based filters

Manual verification of the 36 h dataset from validated dataset identified 4,235 click trains. When the same dataset was analyzed using the rule-based filter, 7,734 pulse events were detected, of which 4,247 were classified as click trains. The rule-based filter correctly detected almost 100% of the click trains identified by manual verification. However, 45% of the detected events were FP.

Similarly, manual verification of a 408 h dataset identified 532 vessel noise events. The rule-based filter detected 2,695 pulse events in this dataset, including 500 true vessel noise events. Thus, the detection rate was 94%, but 81% of the detected events were FP.

### Performance of machine learning model

**Fig. 4:**
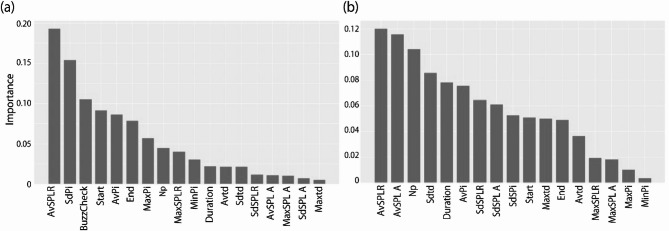
Relative importance of each feature used in the machine learning model for classifying pulse events into (**a**) regular clicks, buzzes, and noise, and (**b**) vessel noise and nnon-vessel noise events. Feature abbreviations are defined in Table 3.

The machine learning model for classifying pulse events into regular clicks, buzzes, and noise was trained and validated using a 72 h dataset comprising 4,868 pulse events. Manual verification identified 1,271 regular clicks, 319 buzzes, and 3,278 noise events. The model achieved an accuracy of 97%, precision of 94%, recall of 96%, F1-score of 95%, and FPR of 2.8% (Table 4). Among the 18 feature values, AvSPLR was the most important, followed by SdPi, BuzzCheck, and Start (Fig. 4a).

**Table 4:** Classification performance of machine learning model for click trains and vessel noise.

Metric	Click train model (%)	Vessel noise model (%)
Accuracy	97	99
Precision	94	99
Recall	96	88
F1-score	95	93
False positive rate	2.8	0.1

The machine learning model for classifying pulse events into vessel noise and noise was trained and validated using a 408-h dataset comprising 3,353 pulse events. Manual verification identified 201 vessel noise and 3,152 other noise events. The model achieved an accuracy of 99%, precision of 99%, recall of 88%, F1-score of 93%, and FPR of 0.1% (Table 4). The most important feature values were AvSPLR, AvSPL A, Np, and Sdtd (Fig. 4b).

To evaluate the efficiency of the developed models, we compared the time required to analyze 395 h of A-tag data. Manual inspection took approximately 36 h, whereas the automated method completed the same analysis in about 1.5 min.

### Validation of the developed algorithm on test data

The performance of the rule-based filter and machine learning model was evaluated using a test dataset. For click train classification, a 16-h dataset comprising 395 manually verified click trains (351 regular clicks and 44 buzzes) was used. When the rule-based filter was applied to the test dataset, 685 pulse events were detected, including all 395 manually verified click trains, resulting in a detection rate of 100%. However, 42% of the detected events were FP. When these events were subsequently classified by the machine learning model, it achieved an accuracy of 83%, precision of 95%, recall of 75%, F1-score of 84%, and FPR of 5.9% (Table 5).

**Table 5:** Classification performance of the machine learning model for click trains and vessel noise on a test dataset.

Metric	Click train model (%)	Vessel noise model (%)
Accuracy	83	67
Precision	95	100
Recall	75	15
F1-score	84	26
False positive rate	5.9	0.0

In contrast, for vessel noise classification, a 456-h dataset was manually verified, identifying 68 vessel noise events. The rule-based filter detected 169 pulse events in this dataset, including 66 true vessel noise events, resulting in a detection rate of 97%. However, 61% of the detected pulse events were FP. When the detected pulse events were classified by the machine learning model, the model achieved an accuracy of 67%, precision of 100%, recall of 15%, F1-score of 26%, and FPR of 0.0% (Table 5). This test dataset contained only 66 true vessel noise events, which likely contributed to the low FPR.

## Discussion

The rule-based filter and the machine learning model were separately developed in this study, and a combined approach was proposed for efficiently detecting click trains and vessel noise recorded by A-tags. The rule-based filter achieved high detection rates: almost 100% for click trains and 94% for vessel noise in the training dataset. Furthermore, when applied to the test dataset with different background noise levels^25^ and finless porpoise populations^24^, it maintained strong performance, detecting 100% of click trains and 97% of vessel noise events. These results suggest that the rule-based filter functions as a robust preprocessing tool for detecting target events under varying site conditions. The primary advantage of this approach lies in its ability to eliminate large amounts of noise while reliably capturing nearly all target events, thereby enhancing analytical accuracy and computational efficiency. However, a substantial proportion of the pulse events detected by the rule-based filter were FP: 45% for click trains and 81% for vessel noise in the training dataset and 42% and 61% in the test dataset. The large number of high FP was largely attributed to broadband impulsive noise produced by snapping shrimp. These pulses often exhibit similar amplitude to click trains and vessel noise, making them difficult to distinguish using rule-based criteria alone. This residual noise was a result of the intentionally relaxed detection criteria, designed to minimize missed detections of true events. Consequently, FP was tolerated under the assumption that they would be removed during manual verification or by the subsequent machine learning classification step. Therefore, for accurate and efficient detection of click trains and vessel noise, combining the rule-based filter with a machine learning model is essential.

The machine learning model for classifying click trains demonstrated high performance on the training dataset (Table 4). The low FPR of 2.8% indicates that the model effectively distinguishes click trains from noise. Although performance declined moderately when applied to the test data, the model still correctly classified 83% of the events (Table 5). Comparable levels of classification accuracy have been reported in previous cetacean acoustic studies using full waveform data. For example, Zahn et al.^45^ reported accuracy of 98% for narwhals (*Monodon monoceros*) and belugas (*Delphinapterus leucas*), while Griffiths et al.^43^ reported 97% accuracy for Dall’s porpoises (*Phocoenoides dalli*) and Kogia spp. However, these studies evaluated performance using only data from the same sites used in model training. In contrast, the present study not only demonstrated similarly high accuracy on the training dataset (Table 4), but also demonstrated the model’s generalizability by testing it on independent data from a different site. This approach provides a clear indication of its robustness for practical application (Table 5). In addition, Song et al.^46^ achieved 93% accuracy in detecting click trains of the Yangtze finless porpoise using a Hilbert–Huang transform combined with a backpropagation neural network, a result comparable to the present study.

While direct comparisons are limited by methodological differences^47,48^, the performance of the model in this study is broadly comparable to those of previous approaches using full waveform data. Notably, the A-tag employed here achieved high classification accuracy despite lacking direct frequency information, which is often regarded as a critical feature in such models^43,45^. The comparison of SPL at two specific frequencies, i.e., SPLR, suggest that it can serve as an effective proxy for frequency information. Phocoenidae produce similar narrow-band high frequency click^49^, and therefore, SPLR is not expected to differ significantly among species. Accordingly, the method developed in this study is likely to be applicable to other Phocoenidae species with only minor adjustments and fine-tuning. Further research is needed to validate its effectiveness for different target species.

In the machine learning model for classification of click trains, the feature values “Start” and “End” showed relatively high importance. As these parameters represent temporal information, their contribution may reflect seasonal and diel behavioral patterns, as well as temporal autocorrelation in their acoustic activity. For instance, finless porpoises in the Kanmon Strait, located at the western entrance of the Seto Inland Sea, are reported to occur mainly at night^19^, suggesting that such a nocturnal activity pattern may have been implicitly learned by the model. Although investigating porpoise behavior was not the primary aim, incorporating this information into the design of the rule-based filter and machine learning model can enhance model accuracy, enabling the development of species-specific classifiers informed by expert knowledge.

The decrease in accuracy for classification of click trains when applying the model to the test dataset may be attributed to several factors. Indeed, significant differences in source level, -3 dB bandwidth, click duration, and the number of clicks per click train have been reported between regular clicks recorded in Mikawa Bay and the Seto Inland Sea^25^. Despite these acoustic differences, the machine learning model maintained high accuracy (Table 5), supporting its applicability to different populations. Moreover, because the A-tag does not record frequencies below 55 kHz, it is less affected by low-frequency background noise, likely resulting in stable detection performance even at sites with varying background noise levels. Thus, the combined use of the rule-based filter and machine learning model is expected to efficiently process large-scale, long-term monitoring datasets.

Vessel noise recorded by the A-tag primarily consisted of ultrasonic components that are audible to finless porpoises^34,34,36,37^. Our method enabled efficient detection of both click trains and vessel noise events from the same dataset, facilitating assessments of acoustic impacts on finless porpoises. The accuracy of the machine learning model for classifying vessel noise was high for the training dataset (Table 4). However, accuracy declined on the test dataset (Table 5), likely due to site-specific differences in vessel noise characteristics, such as vessel type, size, and speed^50–54^, and variation in background noise that can influence signal masking. The test dataset included a relatively small number of vessel noise events, which may have limited the robustness of the evaluation. In particular, the vessel noise classification model exhibited perfect precision but very low recall, suggesting that the model may have learned overly strict classification criteria, thereby failing to detect many true vessel noise events. Although vessel type and speed were not visually assessed, the performance drop in classification suggests major site-specific differences in vessel noise characteristics. The training site in Mikawa Bay is near a busy ferry route and fishing port with frequent small-vessel traffic, whereas the Seto Inland Sea test site is used mainly by large vessels and has fewer fishing boats. The low recall observed in the test data may also have resulted from overfitting to the acoustic features specific to the training environment. In particular, the training data included frequent broadband impulsive noise from snapping shrimp, resulting in a higher background noise level than the test data^25^. This environmental contrast may have led the model to overfit to site-specific acoustic features, resulting in reduced recall on the test dataset. To improve generalization, it is essential to train the model using a more diverse dataset that includes vessel noise recorded under varying conditions, such as different sites, background noise levels, and vessel types. In addition, incrementally updating the model with newly collected data may help develop a more robust machine learning model that can adapt to site-specific differences in acoustic characteristics.

Combining the rule-based filter and machine learning model developed in this study significantly reduced analysis time compared to manual analysis. The combined approach, which first removes noise using the rule-based filter and then applies a machine learning model, achieved over 90% accuracy in detecting click trains and vessel noise on the training dataset. Furthermore, when applied to data from different sites not included in the model development, the method maintained relatively high accuracy for detecting finless porpoise click trains, demonstrating its generalizability. Further research is needed to evaluate the generalizability of the proposed detection method under various background noise conditions and among different populations of finless porpoises. This method was specifically designed for the A-tag. However, the rule-based filter using the pulse intervals, number of clicks in a click train and the SPLR to separate Phocoenidae out of Delphinidae can be applied to other pulse event recorders. The unique feature of A-tag is to separate sound source by the time difference of arrival between two hydrophones. Criteria based on time difference of arrival information provide the rule-based filter with an additional function of source separation. Therefore, although the filter is specifically optimized for the A-tag system, its core framework can be adapted to other devices with suitable modifications. The proposed approach in this study provides a scalable framework for ecological studies of finless porpoises, vessel noise impact assessments, and broader applications in passive acoustic monitoring of other small cetacean species and anthropogenic noise.

## Supplementary Information

Below is the link to the electronic supplementary material.

Supplementary Material 1

## Data Availability

The rule-based filter and model are currently being prepared for use on the website. The data used is available from the corresponding author upon reasonable request.
