# Supplementary material for: A hybrid method combining rule-based filter and machine learning to detect porpoise and vessel sounds from a pulse event recorder
Source: Sci Rep. 2025 Aug 25;15:31211. doi: 10.1038/s41598-025-16370-1 (PMC12379293; doi:10.1038/s41598-025-16370-1)
Supplement: Supplementary file 1 — Supplementary Material 1 [file 41598_2025_16370_MOESM1_ESM.docx]

**Supplementary information**

**Table S1. Information on recording sites and periods.** The recording periods indicate the months and years during which recordings were conducted for at least five days per month. The data used to evaluate the accuracy of the rule-based filter and to train and validate the machine learning model were summarized for each site. For sites St. 1–10 (corresponding to St. M1–M10), data were collected in Mikawa Bay, Japan. These ten sites were selected to encompass a variety of acoustic environments within Mikawa Bay, with the aim of enhancing model robustness against diverse background noise conditions. Data from St. M1–M10 were randomly split into 70% for training and 30% for validation. In contrast, data from site St. S, located in the Seto Inland Sea, were used exclusively for testing and were not included in model training. For finless porpoise click train events, only the first 10 minutes of each hour (totaling four hours per day) were analyzed, whereas vessel noise was analyzed over the entire 24-hour period.

| Monitoring site | Latitude  Longitude | Recording period | Test and training data  (finless porpoise) | Test and training data (vessel sounds) |
| --- | --- | --- | --- | --- |
| St. M1 | 34°43′16.29″N  137°00′44.31″E | Oct–Dec, 2016;  Jan–Apr, 2017;  Sep–Dec, 2018;  Nov–Dec, 2019;  Jan–Mar, Sep–Dec, 2020;  Jan–Mar, Sep–Dec, 2021;  Jan–Mar, Sep–Dec, 2022;  Jan–Mar, 2023 | **Rule-based filter**  Nov 16, 2016;  Feb 16, 2017;  Apr 21, 2019;  Oct 21, 2021  **Machine learning**  Nov 16, 2016;  Feb 16, 2017;  Apr 21, 2019;  Oct 21, 2021 | **Rule-based filter**  Feb 22–26, 2019  **Machine learning**  Feb 22–26, 2019;  Jan 23–27, 2023 |
| St. M2 | 34°42′57.78″N  137°00′19.95″E | Oct–Dec, 2013;  Jan–Apr, Nov–Dec, 2014;  Jan–Mar, Oct–Dec, 2015;  Jan–Mar, 2016;  Sep–Dec, 2022;  Jan–Feb, 2023 | **Machine learning**  Nov 5, 11, 2015;  Feb 5, 2023 | **Machine learning**  Oct 5–8, 2013;  Feb 24–28, 2015;  Mar 1–3, 2015 |
| St. M3 | 34°42′44.10″N  136°59′59.40″E | Oct–Dec, 2016;  Jan–Apr, 2017 | **Machine learning**  Nov 26, 2016 |  |
| St. M4 | 34°42′55.92″N  136°59′39.78″E | Jan–Feb, Aug–Dec, 2020;  Jan–Mar, Oct–Dec, 2021;  Jan–Mar, 2022 | **Rule-based filter**  Jan 4, 2020  **Machine learning**  Jan 4 and Aug 19, 2020 | **Machine learning**  Aug 13, 2020 |
| St. M5 | 34°42′23.10″N  136°59′47.64″E | Aug–Dec, 2020;  Jan–Mar, Oct, Dec, 2021;  Jan–Feb, Sep–Dec, 2022;  Jan–Mar, Oct–Dec, 2023 | **Machine learning**  Jan 27, 2023 | **Rule-based filter**  Nov 14–16, 2022  **Machine learning**  Nov 14–16, 2022 |
| St. M6 | 34°42′10.08″N  136°59′31.20″E | Nov–Dec, 2016;  Jan–Apr, 2017 | **Rule-based filter**  Dec 16, 2016  **Machine learning**  Dec 16, 2016 |  |
| St. M7 | 34°42′10.08″N  136°59′26.82″E | Oct–Dec, 2016;  Jan–Apr, 2017 | **Machine learning**  Feb 4, 2017 |  |
| St. M8 | 34°41′47.79″N  136°59′33.60″E | Oct–Dec, 2013;  Jan–Mar, Nov–Dec, 2014;  Jan–Mar, Nov–Dec, 2015;  Jan–Mar, 2016;  Sep–Dec, 2018;  Jan–Mar, Sep–Dec, 2019;  Jan–Mar, Jul–Dec, 2020;  Jan–Dec, 2021;  Jan– Dec, 2022;  Jan–Dec, 2023 | **Rule-based filter**  Nov 26 and Jul 14, 2022  **Machine learning**  Nov 26, 2013;  Jul 14, 2022 | **Rule-based filter**  Nov 23–24, 2013;  Jan 12–17, 2016;  Mar 22, 2021 |
| St. M9 | 34°41′38.52″N  136°59′45.75″E | Nov–Dec, 2016;  Jan–Mar, 2017;  Nov–Dec, 2019;  Jan, Mar, Sep–Dec, 2020;  Jan–Mar, 2021;  Sep–Dec, 2022;  Jan–Mar, Oct–Dec, 2023 | **Rule-based filter**  Oct 2, 2016  **Machine learning**  Oct 2, 2016;  Nov 12, 2022 | **Machine learning**  Dec 18–19, 2022;  Jul 28, 2023;  Feb 13, 2017 |
| St. M10 | 34°41′51.42″N  137°00′00.30″E | Oct–Dec, 2015 | **Machine learning**  Nov 5, 2022 |  |
| St. S | 33°51′9.10″N  132°6′53.60″E | Jul–Sep, 2021;  Feb, Apr–Dec, 2022;  Jan–Dec, 2023 | **Rule-based filter**  **Machine learning**  Jul 22, 2021;  Sep 26, 2022;  Apr 19 and 28, 2023 | **Rule-based filter**  **Machine learning**  Jul 7, 2021;  Aug 5–12 and Sep 23–24, 2022;  Mar 2–9, 2023 |

**Supplemental Methods**

**A-tag settlements**

Two models of A-tags were used: ML200-AS2 and ML200-AS8. Although both models acquire similar data, the ML200-AS8 has four times higher sound pressure resolution compared to the ML200-AS2. To ensure consistency in data resolution between the two models, sound pressure values recorded by the ML200-AS8 were scaled down by a factor of four.

Additionally, two A-tag configurations were employed: a T-type, in which the hydrophones were arranged horizontally, and an I-type, in which they were arranged vertically (Figure S1). The two hydrophones were separated by 590 mm in the T-type and by 190 mm in the I-type. The T-type A-tag (ML200-AS2) was used from 2013 to 2017 at St. 1–10, while the I-type A-tag (ML200-AS2, ML200-AS8) was used from 2016 to 2023 at St. 1–10. At St. S, the I-type (ML200-AS8) was exclusively used. All data were analyzed using the same procedures regardless of the A-tag configuration or version.

Each A-tag was deployed by suspending it from a buoy tethered by a rope, maintaining hydrophone A at a depth of 3 m in both configurations. In the T-type configuration, hydrophone A was positioned to the south and hydrophone B to the north. In the I-type configuration, hydrophone B was oriented vertically downward.





Figure S1. Two configurations of A-tags and frequency response characteristics of hydrophone A and B. (a) T-type A-tag with horizontally arranged hydrophones. (b) I-type A-tag, with vertically arranged hydrophones. (c) Frequency response curves for hydrophones A (black line, 130 kHz peak) and B (gray line, 70 kHz peak). Panel (c) was adapted from Figure 1 of Kameyama et al.^1^.

**Reference**

1. Kameyama, S. et al. Acoustic discrimination between harbor porpoises and delphinids by using a simple two-band comparison. *J. Acoust. Soc. Ame.*, **136**(2), 922–929 (2014).

.
